# Advanced Polyamidoamine Hydrogels for the Selective Cleaning of Artifacts in Heritage Conservation

**DOI:** 10.3390/polym17192680

**Published:** 2025-10-03

**Authors:** Elisabetta Ranucci, Jenny Alongi

**Affiliations:** Dipartimento di Chimica, Università degli Studi di Milano, via C. Golgi 19, 20133 Milan, Italy; elisabetta.ranucci@unimi.it

**Keywords:** composite hydrogel, polyamidoamine, cleaning, ink, cellulosic artifacts

## Abstract

A polyamidoamine-based hydrogel (H-M-GLY) and its montmorillonite-based composite (H-M-GLY/MMT) were studied as selective cleaning materials for cultural heritage conservation. H-M-GLY was synthesized from a glycine-based polyamidoamine oligomer with acrylamide terminals (M-GLY) through radical polymerization at pH 7.3 and had a basic character. The M-GLY oligomer was in turn synthesized from N,N′-methylenebisacrylamide and glycine in a 1:0.85 molar ratio. H-M-GLY/MMT was obtained by cross-linking a 1:0.1—weight ratio—M-GLY/MMT mixture at pH 4.0, to promote polyamidoamine-MMT interaction. The composite hydrogel absorbed less water than the plain hydrogel and proved tougher, due to montmorillonite’s electrostatic interactions with the positively charged M-GLY units. Scanning electron microscopic analysis showed that MMT was uniformly dispersed throughout the hydrogel. Both hydrogels were subjected to ink bleeding tests on papers written with either iron gall or India ink. Microscopic observation revealed neither bleeding nor release of hydrogel fragments. Being basic, H-M-GLY successfully deacidified the surface of aged paper. H-M-GLY/MMT, swollen in a 1:9 ethanol/water solution, was found to be effective in removing wax, known to trap carbonaceous particles and form dark stains on artistic artifacts. This study demonstrates the great potential of polyamidoamine-based hydrogels as versatile selective cleaning systems for cellulosic and other cultural heritage materials.

## 1. Introduction

The protection, preservation, and transmission of cultural heritage constitute a vital mission for humanity, as it reflects both historical legacy and societal development. In this context, surface cleaning represents a crucial initial step in the restoration and conservation of artwork, preceding both consolidation and surface protection [1]. These processes are essential, as deterioration often begins at the surface and can advance inward, potentially threatening the integrity of the entire piece. Historically, surface cleaning primarily relied on pure solvents combined with mechanical action [2]. However, this approach presents two major drawbacks: solvents can infiltrate deeper layers of the artifact and pose health and environmental risks. Mechanical methods, meanwhile, are often unsuitable for delicate materials [3]. Consequently, there is a growing shift toward alternative techniques, including biocleaning [4], micellar solutions [5,6] and gels [7,8,9] that are gentler, more selective, and better at preserving surface integrity. These techniques offer numerous benefits, as they can be tailored to the chemistry of specific artifacts—such as varnishes, stones, easel or wall paintings, or oxidized metals. They minimize the need for harsh solvents, enhancing safety for both conservators and the environment, and are designed to remove contaminants without damaging original materials [10].

Micellar solutions, for example, are water-based self-assembling systems that encapsulate hydrophobic contaminants while leaving surrounding hydrophilic materials unaffected [11]. Their low surface tension enables gentle penetration into porous substrates without mechanical abrasion, and they can usually be rinsed with minimal residue. Their formulations are highly tunable to target different soils or coatings. However, they provide limited spatial control: being liquid dispersions, they may spread or penetrate sensitive substrates, risking swelling, leaching, or uneven cleaning. Residual surfactants can also remain on the surface, potentially causing long-term issues such as yellowing or stickiness.

Gels, by contrast—whether water- or solvent-based—offer highly localized application, making them ideal for treating specific areas without affecting adjacent zones [12]. They are particularly useful when prolonged contact is required, as they retain the cleaning agent at the surface, allowing gradual release and reducing the risk of overcleaning or leaching. Physical gels have been extensively used by conservators for their ease of application, cost-effectiveness, and reusability their tendency to leave residues remains a concern [9,12]. In contrast, chemical gels typically exhibit greater dimensional stability than physical gels and are less likely to crumble or leave residues.

Gels also prevent dripping, which is advantageous for vertical surfaces and delicate layers, and they reduce the need for scrubbing or mechanical action [9]. In practice, gels enable non-invasive cleaning, allow pH control, and can assist in surface consolidation, though sometimes at the cost of lower raw cleaning efficiency. Their versatility has been expanded using targeted agents, such as enzymes, which enhance specificity and reduce application times [13]. Even greater potential lies in hybrid gel/microemulsion systems, which provide conservators with a powerful yet controlled cleaning tool [14]. These systems combine the solubilizing power of micelles or emulsions with the spatial precision of gels, avoiding the risks of uncontrolled liquid penetration. As a result, they are particularly valuable for the treatment of fragile, porous, or irreplaceable cultural heritage materials.

Paper artworks are highly vulnerable because cellulose is prone to acid hydrolysis, oxidation, and depolymerization, which progressively weaken paper [15]. Acidity also promotes biodeterioration, as most fungi thrive under slightly acidic conditions [16]. To counter these effects, deacidification is essential to prevent cleavage of cellulose β-glycosidic bonds and to enhance resistance against both chemical and biological deterioration. Various strategies have been developed to introduce an alkaline reserve, using both aqueous and non-aqueous systems [17]. More recently, hydrogels have been explored for mild acid removal in aged papers, sometimes combine cleaning and buffering, as in nano-CaCO_3_ loaded hydrogels [18] or gellan gum matrices with carboxymethyl cellulose–stabilized nano-Mg(OH)_2_ dispersions [19]. The choice of method depends on the conservation state of the paper, and the sensitivity of graphic media to solvents.

Wax cleaning is also essential in cultural heritage preservation. Waxes protect artifacts by forming a thin barrier on surfaces such as wood, metal, or stone, shielding them from dust, moisture, and pollutants that cause corrosion or decay [20]. Removing degraded waxes and residues prevents harmful chemical reactions that could damage the underlying material. This is particularly important for paintings exposed to candle vapors in churches, where wax accumulation can harm wood and canvas. Wax removal is normally performed with the help of solvent mixtures [21].

Polyamidoamines (PAAs) are synthesized by the aza-Michael polyaddition of bis-acrylamides with bis-secondary- or primary amines, including α-amino acids [22]. They can be prepared in concentrated aqueous solutions, at room temperature, and without the addition of catalysts that align with green chemistry principles [23]. PAAs can be synthesized for being biocompatible and biodegradable and, therefore, find use in biotechnological applications, for instance as drug carriers [24] or non-viral vectors for gene therapy [25]. One of the distinctive features of α-amino acid-derived PAAs is their eco-compatibility [26,27,28]. Cross-linked PAAs form hydrogels capable of absorbing large amounts of water [29], which have been studied as substrates for tissue engineering [30] and as resins for absorbing heavy metal ions [31]. Tough, compression resistant PAA hydrogels can be obtained by incorporating sodium montmorillonite, which establishes strong interactions with cationic PAAs through adsorption mechanisms [32]. Finally, both linear and cross-linked PAAs have demonstrated thermal stability [33] and resistance to photo-oxidation [34].

Previous studies have highlighted water-soluble PAAs as promising paper preservatives, with their ammonium cationic groups conferring both deacidification capacity, particularly of iron-gall inked paper and biostatic activity [35,36] and for the conservation and enhancement of naturally aged paper [37].

The objective of this study is to show that PAA hydrogels can be precisely engineered as soft sponges for the selective cleaning of delicate historical artifacts. Special attention is given to their use for the gentle deacidification of aged, inked paper and, when formulated as tougher montmorillonite-based composites, as controlled reservoirs of hydroalcoholic solvents for removing wax deposits.

## 2. Materials and Methods

### 2.1. Materials

N,N’-methylenebisacrylamide (MBA, 99%), glycine (GLY, >99%), D_2_O (99.9%), DCl (35% in D_2_O), fuming hydrochloric acid (HCl, ≥37% in water), lithium hydroxide monohydrate (LiOH ⋅ H_2_O, ≥98%), potassium persulfate (>99%) sodium metabisulfite (>99%), sodium montmorillonite Nanoclay Nanomer^®^ (MMT, Sigma-Aldrich, Milan, Italy) and ethanol (96%) were supplied by Sigma Aldrich (Milan, Italy).

The paper used in the cleaning tests were a Calligraphy Canvas paper supplied by Cartotecnica Favini s.r.l. (Rossano Veneto, Italy), density 100 g m^−2^, and a Smooth Drawing Paper A4 supplied by Fabriano (Fabriano, Ancona, Italy), density 220 g m^−2^. The Encre de Chine India Graphic Ink, in black color, supplied by Pebeo © Italia Srl (Milan, Italy), and the Schreibintinte Iron Gall Nut Ink, in ebony color, supplied by Rohrer & Klingner^®^ (Zella-Mehlis, Turingia, Germany) were used.

Wax from a vegetable candle was purchased from Ecofriendly Magic Lights candles, (Grugliasco, Turin, Italy).

### 2.2. Synthesis of the Functionalized M-GLY Oligomer

N,N′-methylenebisacrylamide (9.34 g, 60 mmol), glycine (3.87 g, 51 mmol) and lithium hydroxide monohydrate (2.19 g, 51 mmol) were suspended in distilled water (25 mL) and maintained under stirring at 50 °C until complete dissolution. The reacting solution was then left in the dark at 25 °C for five days. The resulting viscous solution was divided into two equal aliquots: one was acidified to pH 4.0 and the other to pH 7.3 using 6 M aqueous HCl. Both solutions were used without further treatment. A 1 mL aliquot of the pH 4.0 solution was freeze-dried and analyzed by ^1^H-NMR in D_2_O.

### 2.3. Synthesis of the H-M-GLY/MMT Hydrogel

H-M-GLY/MMT hydrogel disks (20 mm diameter × 2 mm thickness) were synthesized in a custom glass mold consisting of two silanized glass plates (2 mm thick each) separated by a 2 mm silicone spacer containing a 20 mm circular opening. The mold was tightly sealed by securing with metal clamps. The glass plates were first cleaned by soaking in aqua regia for 5 h, rinsed thoroughly with water, dried, and then silanized by exposure to trichloromethylsilane vapors in a sealed chamber for 3 days. Following silanization, the plates were washed sequentially with toluene (20 mL), ethanol (2 × 20 mL), and water (3 × 20 mL), and finally dried by gentle wiping.

An aliquot (2 mL) of a 38 wt% M-GLY solution at pH 4.0 was purged with nitrogen. MMT (0.08 g) was then slowly added under stirring. The homogeneous dispersion was stirred overnight under nitrogen, after which sodium potassium persulfate (2 mg) and sodium metabisulfite (1.6 mg) were added. The resulting solution was injected into the glass mold and allowed to react at 25 °C for 24 h. After this period, the cross-linked product was removed from the mold using a stainless-steel spatula, yielding an opalescent, tough, and flexible gel.

### 2.4. Synthesis of the H-M-GLY Hydrogel

Plain H-M-GLY hydrogel samples were prepared from a 30 wt% M-GLY solution at pH 7.3, obtained by diluting the solution described in Section 2.2. The same procedure used for preparing H-M-GLY/MMT hydrogels was followed, except that MMT was omitted.

### 2.5. Chemical Characterization

The chemical structure of the M-GLY oligomer was assessed by ^1^H-NMR, collecting spectra in D_2_O at pH 4.0 and at 25 °C using a Bruker Advance DPX-400 NMR spectrometer (Milan, Italy) operating at 400.13 MHz. Parameters: scan number 32, relaxation delay, d1, 10.0 s, receiver gain automatically measured and set by the instrument.

H-M-GLY, H-M-GLY/MMT and MMT were analyzed by attenuated total reflectance (ATR) Fourier-transform infrared spectroscopy (FT-IR). FT-IR/ATR spectra were recorded at room temperature, in the 4000–600 cm^−1^ wavenumber range, with 64 scans and 8 cm^−1^ resolution, using a Jasco FT-IR/FIR spectrophotometer (Milan, Italy), equipped with a diamond crystal (1.66 mm penetration depth).

The surface pH of the aged filter paper samples, both untreated and treated with the H-M-GLY hydrogel, was measured with a surface PH 7 Vio^®^ pH-meter (Steroglass, Perugia, Italy) equipped with a flat electrode. Prior to measurements, the electrode was calibrated with pH 4.00 and pH 7.00 standard solutions. For each paper strip, pH was measured at three different points after adding ultrapure water (10 μL) to the surface, and the electrode was then placed in perpendicular contact with the wet spots.

### 2.6. Water Uptake Test

Water uptake tests were performed on H-M-GLY and H-M-GLY/MMT hydrogel samples. Hydrogel disks (20 mm diameter × 2 mm thickness), were first immersed in water for 4 h to reach equilibrium swelling and then dried over CaCl_2_, for 24 h. The water uptake was calculated using Equation (1):

(1) $$Water\;uptake\;\%\;=\frac{W_{t}-W_{0}}{W_{0}}\times 100$$

where *W*_0_ = weight of the dry cotton strip and *W_t_* = weight of the hydrated cotton strip. Four hydration/drying cycles were performed.

Each test was performed in five independent replicates.

### 2.7. Morphological Analysis

The fracture surfaces of a dry H-M-GLY/MMT sample (5 mm × 5 mm) were platinum metallized and then analyzed with a Zeiss field-emission scanning electron microscope (FE-SEM), model ZEISS-SIGMA 300 operating at 8.5 mm working distance and 5 kV beam voltage (Zeiss, Ramsey, NJ, USA).

Micrographs of paper samples subjected to ink bleeding tests were obtained using an optical Dino-Lite Edge digital microscope, AM7115MZT model, with 5 MP resolution (VWR International s.r.l., Milan, Italy), operating at 50× magnification.

Micrographs of hydrogel samples subjected to wax cleaning tests were obtained using a digital, fluorescence microscope Dino-Lite^®^ Special Light, AM4115T-GFBW (Taiwan, China) (VWR International s.r.l., Milan, Italy), operating at 50× magnification. Both microscopes were equipped with the DinoXcope, software version 2.6 (2024).

### 2.8. X-Ray Diffraction Analysis

The X-ray diffraction (XRD) spectra of H-M-GLY and H-MGLY/MMT samples were recorded using a Miniflex 600 diffractometer with Cu Kα1 radiation at 1.5405 Å, at 40 kV voltage and 15 mA current of Rigaku Europe SE (Neu-Isenburg, Germany).

The M-GLY/MMT sample used in the XRD analysis were prepared as follows: MMT (0.10 g) was suspended in 10 mL water under stirring for 24 h. After this time, an M-GLY oligomer solution (0.70 g in 10 mL water) was added, the obtained suspension maintained under stirring for 0.5 h. and finally freeze-dried.

### 2.9. Compression Test

Compression tests were performed on H-M-GLY and H-M-GLY/MMT disks (20 mm diameter × 2 mm thickness), which had been pre-soaked at 25 °C for 24 h in water or a 10% aqueous ethanol solution, respectively, to ensure full swelling. While submerged, the samples were subjected to increasing compressive stress using a set of calibration weights (5 g, 10 g, 20 g, 50 g, 100 g and 200 g). Each test was performed in five independent replicates.

### 2.10. Ink Bleeding Test

Ink bleeding tests were carried out on 80 mm × 80 mm squares of written paper, using different combinations of paper and ink. India ink was applied using a round brush with synthetic bristles only on the smooth Fabriano paper. The iron gall ink was applied using a fountain pen (Aurora 88 pen supplied by Aurora, Turin, Italy) on both smooth Fabriano and Calligraphy Canvas Favini paper. Tests were conducted using both H-M-GLY and H-M-GLY/MMT hydrogel samples, which had been immersed in water and 10% aqueous ethanol solution, respectively, for 4 h to reach equilibrium swelling. The hydrogel samples were placed on the inked surface for an initial duration of 45 s, with subsequent tests extending the contact time up to 15 min, and finally gently rubbing. Ink bleeding was evaluated based on the extent of diffusion across the paper and the presence of blurring or spotting. The absence of ink residues on the hydrogels was visually ascertained. Each test was performed in five independent replicates.

### 2.11. Paper Deacidification Test

Paper deacidification tests were carried out on aged filter paper strips (30 mm × 30 mm, Whatman^®^ grade 1, Merck, Milan, Italy) using H-M-GLY hydrogel disks (29 mm diameter × 4 mm thickness) synthesized at pH 7.3, which had been immersed in water for 4 h to reach equilibrium swelling. Filter paper was spot-wetted with water droplets (3 spots, 10 μL each), and the pH was measured using a surface pH meter. A hydrogel disk was placed in contact with the wetted paper spot and gently compressed for 10 s. The pH of the treated spot was then measured. This procedure was repeated in several short intervals (8 × 10 s) within a 10 min test period. Each test was performed in five independent replicates.

### 2.12. Wax Cleaning Test

An H-M-GLY/MMT hydrogel disk (29 mm diameter × 4 mm height) was dried to constant weight under an air draft and then immersed in an aqueous 5% ethanol solution for 4 h until maximum absorption was reached. A wax layer of approximately 45 mg was prepared by dripping molten wax from a burning vegetable candle onto a glass slide and allowing it to solidify. The hydrogel sample was placed on the wax layer for 10 min and then used to gently rub it. Following this treatment, the hydrogel was regenerated by soaking in a 5% aqueous ethanol solution. Three applications were performed, for a total of 60 min of cleaning per glass slide. The test was performed in five independent replicates. A control slide was prepared and cleaned by rubbing with a cotton swab soaked in pure ethanol (>99%).

### 2.13. Durability Test

A two-year-aged dry H-M-GLY/MMT hydrogel disk (29 mm diameter × 4 mm height), which had been prepared as described in Section 2.2, was immersed in a 10% aqueous ethanol solution for 24 h until equilibrium absorption was reached. A wax layer of approximately 8 mg was prepared by dripping molten wax from a burning vegetable candle onto a glass slide and allowing it to solidify. The H-M-GLY/MMT hydrogel sample was used to gently rub the wax layer 8 × 10 s. The test was performed in five independent replicates.

## 3. Results

### 3.1. Synthesis of a,w-Acrylamide Terminated M-GLY Oligomer

**Scheme 1:**
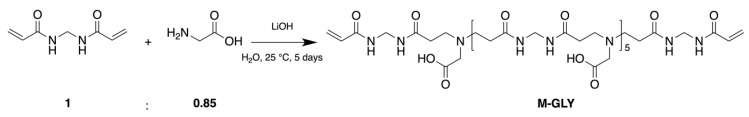
Synthesis of the a,w-acrylamide-terminated M-GLY oligomer.

**Figure 1:**
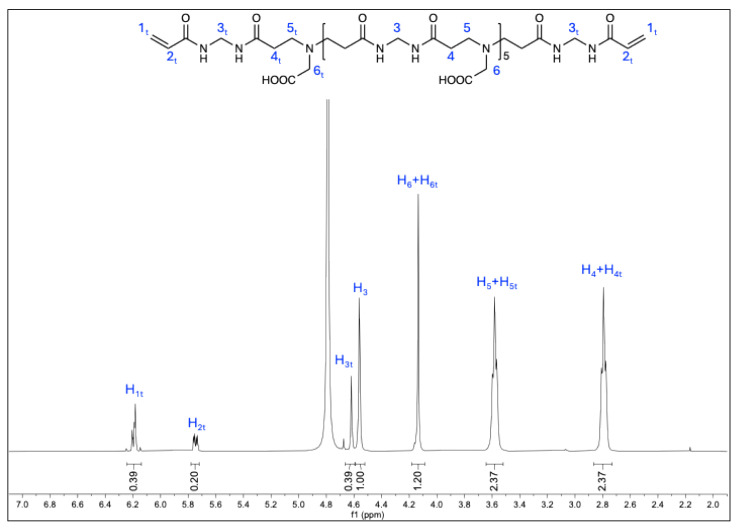
^1^H-NMR spectrum of the a,w-acrylamide-terminated M-GLY in D_2_O at pH 4.0.

An M-GLY polyamidoamine oligomer bearing acrylamide terminals was synthesized through the aza-Michael polyaddition of N,N′-methylenebisacrylamide (MBA) with glycine in a 1:0.85 molar ratio (Scheme 1). One well-established way to tune the degree of polymerization of oligomers is indeed by adjusting the non-stoichiometric ratio between the complementary A and B groups of A-A/B-B monomers [38]. The reaction was conducted under standard experimental conditions, that is, 38 wt% concentration, pH 11, and room temperature [22]. The structure of the M-GLY oligomer was confirmed using ^1^H-NMR spectrometry (Figure 1), showing agreement with the expected molecular structure. The number-average molecular masses (Table 1) were consistent with theoretical values.

(2) $$r=\frac{I_{H6}+I_{H6t}}{I_{H3}+I_{H3t}}$$

where I_H6_ and I_H3_ represent the integrals of internal protons—and I_H6t_ and I_H3t_ those of the terminal protons—of the amine (glycine)-derived and acrylamide (MBA)-derived segments of the repeat units, respectively.

(3) $$n=\frac{I_{H3}}{\frac{I_{H3t}}{2}}$$

(4) $${\bar{X}}_{n}=\left(n+1\right)\times 2+1$$

(5) $${\bar{M}}_{n}=M_{AB}\times \left(n+1\right)+M_{A}$$

where M_AB_ is the sum of the masses of monomers A and B, and M_A_ is the mass of monomer A.

**Figure 2:**
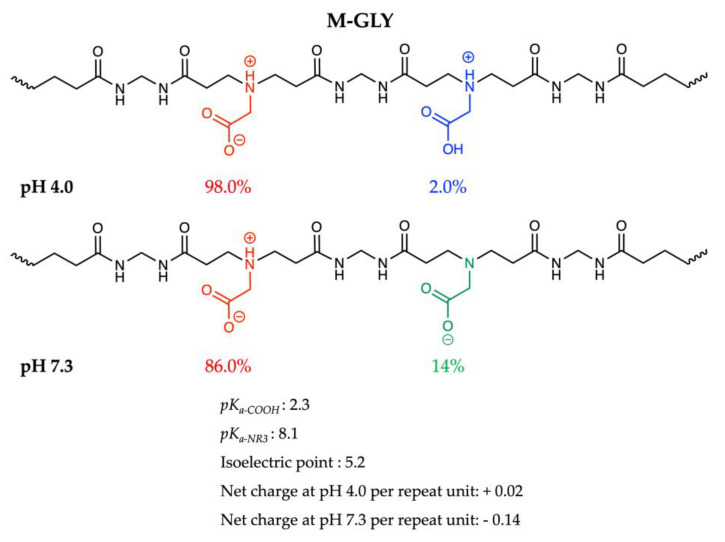
M-GLY ionic species distribution at pH 4.0 and 7.3 [40].

Previous studies had shown that stable complexes form between cationic PAAs and MMT, due to the strong ionic interactions deriving from the exchange reactions involving the cationic groups in PAA repeat units and sodium cations within the MMT interlayer space [32,39]. The M-GLY polymer is an amphoteric PAA bearing carboxyl and tertiary amine groups in its repeat units (Figure 2). As such, it is characterized by distinct pH-dependent ionic species distributions that govern the interaction mode with MMT. The ionic species distribution of M-GLY [40], obtained from the *pK_a_* values of the ionizable functions present listed in Figure 2, indicates that at pH 4.0 its repeat units bear an overall positive charge. Therefore, the complexation with MMT was performed at this pH.

H-M-GLY/MMT composite hydrogel samples were prepared by dispersing MMT in a 38 wt% aqueous MGLY solution at pH 4.0, using a 1:0.1 M-GLY/MMT weight ratio, and then triggering the polymerization of the M-GLY acrylamide terminals with a redox initiator. Plain H-M-GLY hydrogel samples were prepared following the same general procedure but starting from a 30 wt% aqueous MGLY solution at pH 7.3 and in the absence of MMT. The aim was to obtain softer hydrogels suitable for the deacidification of paper, a particularly delicate substrate.

**Figure 3:**
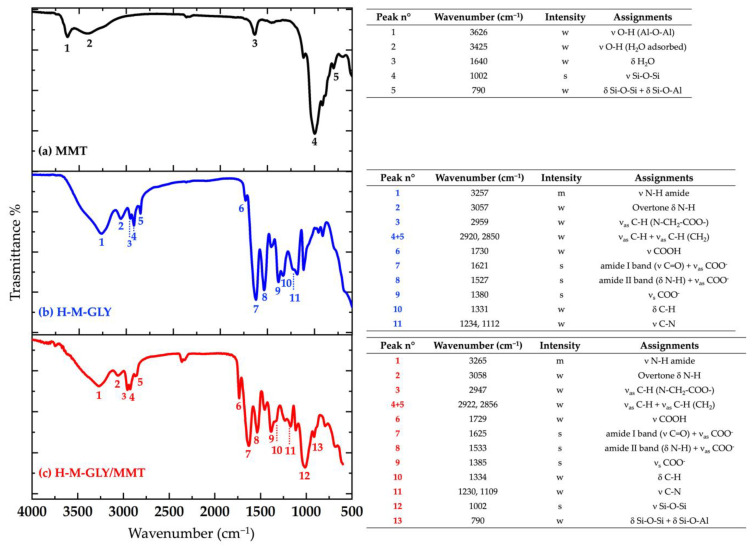
FT-IR/ATR spectra of MMT (**a**), H-M-GLY (**b**) and H-M-GLY/MMT (**c**).

All hydrogels were analyzed using FT-IR/ATR spectroscopy (Figure 3). The spectra were compared to the MMT spectrum, which shows the typical vibrations of a natural smectite with a specific peak at around 1030 cm^−1^ ascribed to Si-O-Si vibration, beyond the typical peaks due to water (Figure 3a). In both H-M-GLY and H-M-GLY/MMT hydrogels, strong absorptions are observed in the 1600–1500 cm^−1^ range, which are attributed to N–C=O stretching vibrations at 1680 cm^−1^, as well as amide N–H bending and C–N stretching from the M-GLY repeat units (Figure 3a,b).

### 3.2. Morphological and X-Ray Diffraction Analyses

**Figure 4:**
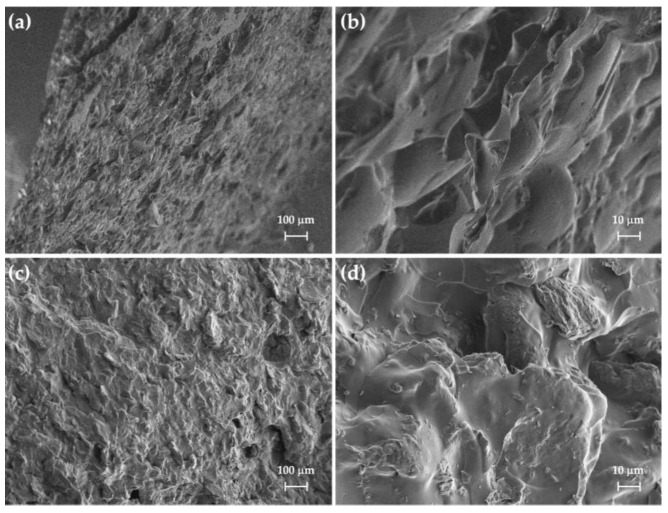
FE-SEM micrographs of H-M-GLY (**a**,**b**) and H-M-GLY/MMT (**c**,**d**) samples. Magnifications: 250× (**a**,**c**) and 2500× (**b**,**d**).

The morphology of a H-M-GLY/MMT sample was observed by FE-SEM (Figure 4a,d) alongside a control H-M-GLY hydrogel sample (Figure 4a,b) observed using the same magnifications. While the H-M-GLY hydrogel appeared characterized by a porous structure due to water evaporation, the H-M-GLY/MMT composite exhibited a compact structure with seamless integration of the hydrogel matrix with montmorillonite. This result was attributed to the strong electrostatic interactions between them [39].

**Figure 5:**
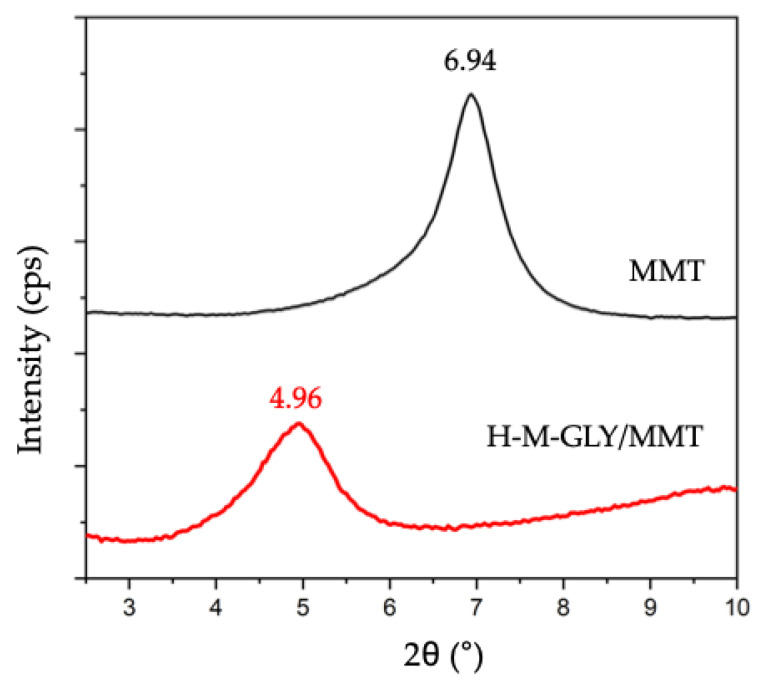
XRD patterns of MMT and H-M-GLY/MMT.

The occurrence of these strong interactions was confirmed by X-ray diffraction (XRD) spectroscopy. The XRD spectra of an M-GLY/MMT complex prepared by mixing the two components in water in a 1:10 weight ratio (Figure 5) showed that the characteristic 001 diffraction peak of MMT at 2Θ = 6.94° shifted to lower angles (4.96°) indicating an increase in the MMT interlamellar spacing from 1.27 nm to 1.78, thus confirming the formation of an intercalated system [39].

### 3.3. Water Uptake Tests

**Figure 6:**
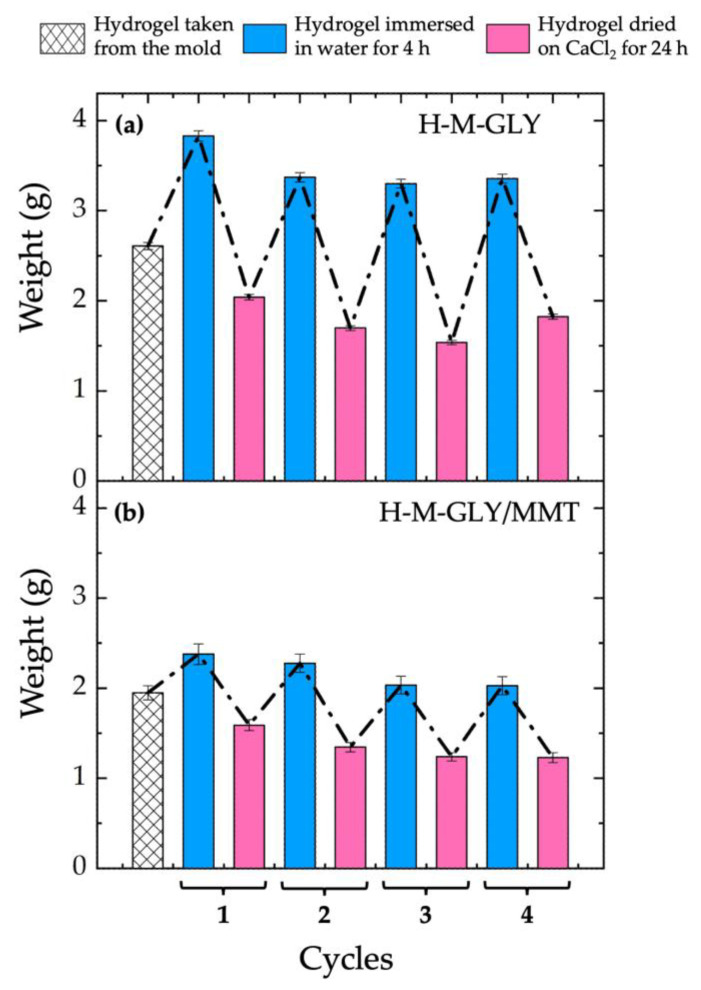
Water uptake of H-M-GLY (**a**) and H-M-GLY/MMT (**b**) hydrogels. Gray bar: sample just extracted from the mold; blue bars: water swollen samples; pink bars: dry sample. Data are the average of five experiments: standard deviation 3%.

To evaluate resistance to osmotic stress—and thus the potential applicability of the hydrogels—four swelling/drying cycles were performed, aimed at measuring the extent of water absorption and subsequent dehydration. A slight decrease in weight was noted at the end of the four cycles for H-M-GLY/MMT, likely due to the loss of soluble fractions trapped within the network. Overall, the H-M-GLY matrix almost doubled its weight during swelling tests, while the H-M-GLY/MMT composite gained from 60 to 80% of its weight on average. Most importantly, data shown in Figure 6 demonstrate that both hydrogels have excellent 3D stability in repeated water absorption and desorption over time. This characteristic makes them strong candidates for water or aqueous solution absorption, without compromising their structure or intended functionality.

### 3.4. Compression Tests

**Figure 7:**
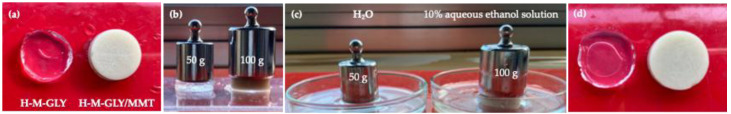
Pictures of H-M-GLY and H-M-GLY/MMT hydrogels: (**a**) before test, (**b**) under instantaneous stress, (**c**) under 1 h stress in water for H-M-GLY and 10% aqueous ethanol solution for H-M-GLY/MMT, and (**d**) after the compression test.

Compression tests were performed on H-M-GLY and H-M-GLY/MMT hydrogels in their fully swollen state. H-M-GLY samples were swollen in water, while H-M-GLY/MMT samples were swollen in 10% aqueous ethanol. All H-M-GLY samples remained apparently intact when compressed by a 50 g weight and showed the first signs of structural deformation under a 100 g weight. As expected, H-M-GLY/MMT samples exhibited greater resistance to compression than the plain hydrogel, remaining unaltered under a 100 g (Figure 7) and deforming only under a 200 g weight. These data, though preliminary, suggest that the compression strength of H-M-GLY was at least 0.06 Kg cm^−1^—around 7 KPa—and that of H-M-GLY/MMT was at least 0.12 Kg cm^−1^—around 14 KPa.

### 3.5. Ink Bleeding Tests

Ink bleeding refers to the unwanted spreading of ink on substrates, typically triggered by the presence of solvents or water. Paper substrates are particularly prone to this phenomenon, as such fluids can enhance ink diffusion. For hydrogels intended for artwork cleaning, a critical requirement is minimal to no interaction with either the ink or the substrate to preserve their integrity during treatment [41].

**Figure 8:**
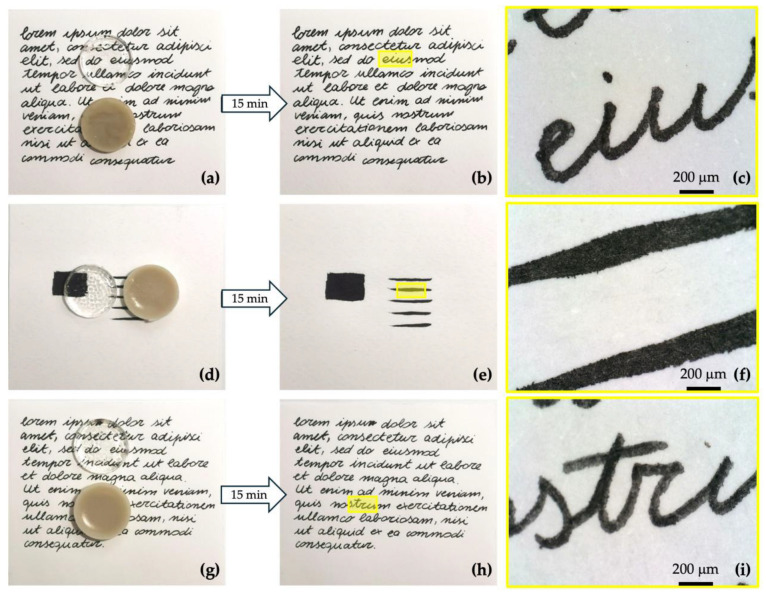
Results of the bleeding tests performed on: Calligraphy Canva FAVINI paper written with iron gall ink (**a**–**c**) and India ink (**d**–**f**); smooth Fabriano paper written with iron gall ink (**g**–**i**). Images represent the appearance of the written samples before (**a**,**d**,**g**) and after prolonged contact (**b**,**e**,**h**) with H-M-GLY and H-M-GLY/MMT samples. Optical micrographs (**c**,**f**,**i**) were taken at 50×.

**Figure 9:**
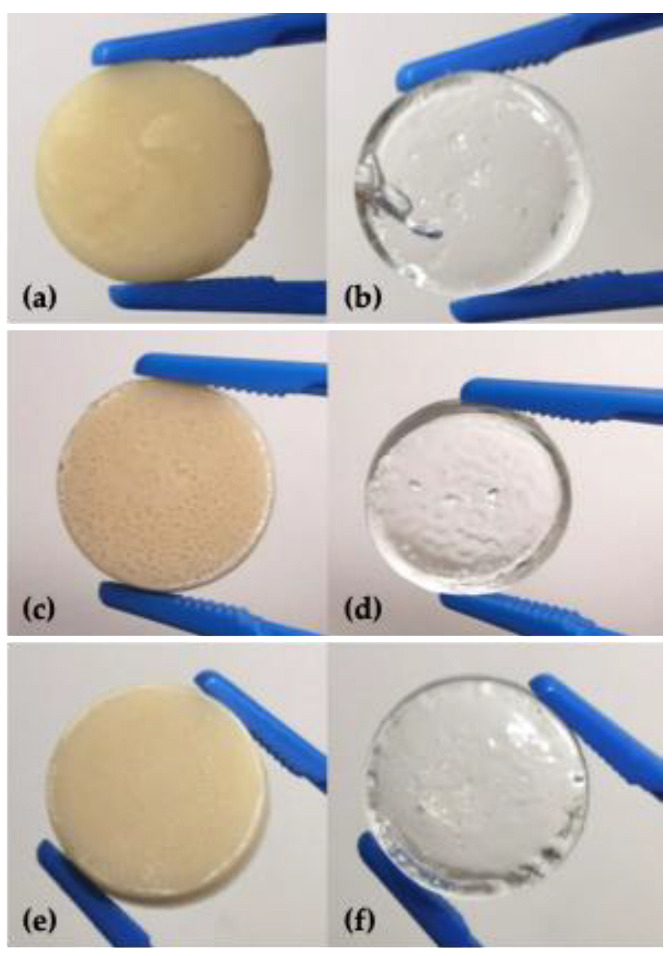
Hydrogel samples after contact with: FAVINI Canvas paper with iron gall ink ((**a**) H-M-GLY/MMT; (**b**) H-M-GLY); FAVINI Canvas paper with India ink ((**c**) H-M-GLY/MMT; (**d**) H-M-GLY); Fabriano Smooth paper with iron gall ink ((**e**): H-M-GLY/MMT; (**f**) H-M-GLY).

In this study, the compatibility between both H-M-GLY and H-M-GLY/MMT hydrogels and written paper was assessed by evaluating ink bleeding after extended hydrogel/paper contact and gentle rubbing. Bleeding tests were performed on marked paper using various combinations of paper types and inks. The objective was to verify that the hydrogels do not interact with two historically significant inks commonly found in manuscript conservation: India ink, used from around 400 AD to the present, and iron gall ink, prevalent from the Middle Ages to the 20th century. Application methods were chosen to reflect historical practices: India ink was applied in solid areas with a brush, mimicking woodblock print techniques (e.g., Japanese ukiyo-e), while iron gall ink was applied with a dip pen to simulate handwriting. No ink bleeding was observed in any of the tested paper samples following application of the hydrogel disks, indicating their compatibility with all tested ink and paper types. This is particularly evident from the analyses shown in Figure 8, which include also images obtained with an optical microscope (Figure 8c,f,i), revealing structural details of the inked paper sheets. Notably, no ink residues were detected on the hydrogel surfaces (Figure 9).

### 3.6. Paper Deacidification Tests

The ability of the H-M-GLY hydrogel to deacidify partially hydrolyzed paper was evaluated using aged filter paper—surface pH 6.22—as a model substrate. Paper sheets were wetted in a controlled manner and gently contacted with H-M-GLY hydrogel disks synthesized at pH 7.3 under mild compression. This procedure was repeated in several short intervals (8 × 10 s), corresponding to a total contact time of 80 s. The hydrogel’s deacidifying effect is demonstrated by the progressive increase in surface pH of paper (Table 2). Its efficacy is attributed to the partially basic character of M-GLY, whose tertiary amine groups in the main chain are ~16% deprotonated at pH 7.3 (Figure 2).

### 3.7. Wax Cleaning

**Figure 10:**
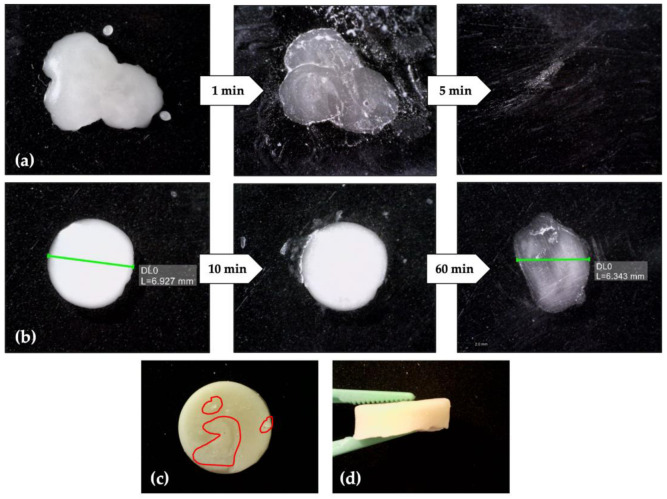
Wax cleaning test: cleaned surface using ethanol-soaked cotton swab (**a**), and H-M-GLY/MMT swollen in a 5% aqueous ethanol solution (**b**); H-M-GLY/MMT after wax cleaning (**c**,**d**). In (**c**), the red lines indicate the presence of wax traces.

Wax cleaning tests were carried out using H-M-GLY/MMT hydrogels fully swollen with a 5% aqueous ethanol solution. Wax stains (45 mg) were deposited on the top of glass slides. As a control, the effect of pure ethanol on removing the wax layer from the glass slide was assessed by rubbing with an ethanol-infused cotton swab. Wax removal using pure ethanol (Figure 10a) required vigorous rubbing for totally 5 min, during which the cotton swab was wetted with ethanol three times. Ethanol partially dissolved, swelled and softened the wax layer, which was largely removed through mechanical action. In the same test, H-M-GLY/MMT hydrogels were applied three times providing a total of 30 min of gel–wax contact and used to gently rubbing. Under the adopted conditions, wax stains were partially removed from the glass, leaving on average 13% residual wax—with a 5% uncertainty (Figure 10b). Wax removal was confirmed by the presence of debris on the gel surface (Figure 10c). At the end of the test, the reinforced hydrogel remained intact, showing no cracks or signs of damage (Figure 10c,d), whereas the plain hydrogel, tested in control experiments, left 27% residue, fractured under the same stress conditions and was not further considered for this application.

### 3.8. Hydrogel Durability

**Figure 11:**
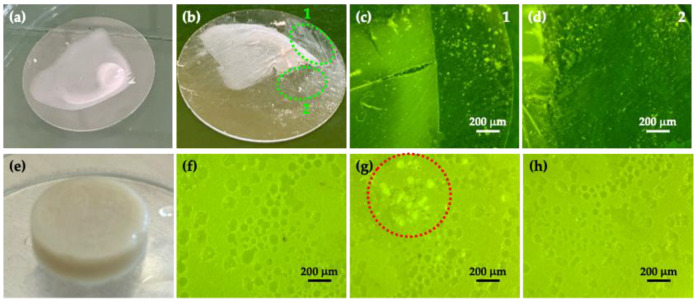
Pictures of wax spot before (**a**) and after applications of the H-M-GLY/MMT hydrogel (**b**). Optical fluorescent micrographs of the wax spot and debris at the edge of the support at points 1 and 2 (**c**,**d**). Picture (**e**) and optical fluorescent micrographs of H-M-GLY/MMT before treatment (**f**); after treatment (**g**), where wax traces are indicated by the red curve; and after washing with a 10% aqueous ethanol solution for 10 s (**h**). Optical micrographs (**c**,**f**,**i**) were taken at 50×.

Both H-M-GLY and H-M-GLY/MMT hydrogels were typically stored at 5 °C for several months in a 5% aqueous ethanol solution, which prevented microbial contamination and mold growth, leaving the hydrogels unaffected. Moreover, as noted in the Introduction, the M-GLY matrix is known for its high thermal and photo-oxidative stability. To assess hydrogel durability under working conditions, a second set of wax-cleaning tests was performed using H-M-GLY/MMT disks aged for two years. The initially dry samples were swollen to equilibrium in a 10% aqueous ethanol solution. The morphology of the aged hydrogels were intact and unchanged compared to freshly prepared samples after swelling. Wax stains (8 mg) were deposited on glass slides, and removal was achieved by gently rubbing using the composite hydrogel for 10 s, followed by cleaning its exposed surface with a cotton swab, then briefly washing in the ethanol solution. This procedure was repeated 8 times. During the experiment, the wax layer progressively faded under the mechanical action exerted by the reinforced hydrogel (Figure 11), and debris was displaced toward the glass border. After each pass, the hydrogel surface was soaked in the 10% aqueous ethanol solution and then gently rubbed to remove residues. Despite repeated treatments, the hydrogel surface remained unaltered throughout the experiment (compare Figure 11f with Figure 11h).

## 4. Conclusions

Gels are three-dimensional networks able to retain fluids and release them in a controlled way. This makes them highly effective for localized, selective cleaning of cultural heritage objects.

We developed polyamidoamine-based hydrogels, both plain and montmorillonite-reinforced, and evaluated their performance as cleaning systems. The gels were synthesized via redox-initiated crosslinking of an acrylamide-terminated, glycine-based polyamidoamine oligomer. The clay-reinforced gel—prepared at pH 4.0 to promote montmorillonite–polyamidoamine interactions—was specifically designed for wax cleaning, as the reinforcement enhances the material’s mechanical resistance. In fact, in wax-cleaning tests, both freshly synthesized and two-year-aged montmorillonite-reinforced gels loaded with aqueous ethanol efficiently removed wax deposits while retaining their morphology and functionality, whereas the plain hydrogel control was considerably less resistant to mechanical stress.

Both gel types demonstrated excellent reversibility in water uptake and did not cause ink bleeding when applied to historically relevant inks and papers.

Moreover, plain soft hydrogels, synthesized at pH 7.3 to enhance their basicity, effectively deacidified aged paper.

These results demonstrate the strong potential of polyamidoamine and polyamidoamine–clay hydrogels as robust, non-invasive, and customizable cleaning tools for cultural heritage conservation. Their structural versatility, which can be obtained with a variety of chemical functions in the repeat units, each characterized by distinct solubilities and/or acid/base behavior, opens the way to tailored systems addressing diverse conservation challenges.

## Figures and Tables

**Table 1:** Molecular mass of the M-GLY oligomer ^a^.

Sample	Theoretical r ^b^ Value	Experimental r Value	n ^c^	${\bar{X}}_{n}$ ^d^	${\bar{M}}_{n}$ ^e^
M-GLY	0.85	0.860	5	13	1500

^a^ Determined through ^1^H-NMR (Figure 1). ^b^ ***r*** = molar monomer ratio in the feed; ^c^ number of the internal repeat units; ^d^ number average polymerization degree; ^e^ number average molecular mass determined using Equations (2)–(5).

**Table 2:** Results of deacidification tests, carried out using aged Whatman Grade 1 filter paper and a plain H-M-GLY hydrogel ^a^.

Paper/Exposure Cycle to H-M-GLY Contact ^b^	pH ^c^
0	6.22
1	6.22
2	6.25
3	6.58
4	6.60
5	6.57
6	6.65
7	6.70
8	6.80

^a^ H-M-GLY pH = 7.3. ^b^ Contact time = 10 s under gentle compression. ^c^ Data represent the average of at least three measurements taken at different locations, with a standard deviation of approximately 2–3%.

## Data Availability

Data are contained within the article.
